# A Procedure to Measure the *in-Situ* Hygrothermal Behavior of Earth Walls

**DOI:** 10.3390/ma7043002

**Published:** 2014-04-11

**Authors:** Pierre-Antoine Chabriac, Antonin Fabbri, Jean-Claude Morel, Jean-Paul Laurent, Joachim Blanc-Gonnet

**Affiliations:** 1 Agence de l’Environnement et de Maitrise de l’Energie, 20 avenue du Grésillé, BP 90406, 49004 Angers Cedex 01, France; 2 Ecole Nationale des Travaux Publics de l’Etat, CNRS-LTDS, UMR 5513, LGCB, 3 rue Maurice Audin, Vaulx-en-Velin, F-69120, France; E-Mails: pierre-antoine.chabriac@entpe.fr (P.-A.C.); jeanclaude.morel@entpe.fr (J.-C.M.); joachim.blancgonnet@entpe.fr (J.B.-G.); 3 CNRS/UJF-Grenoble 1/G-INP/IRD, LTHE UMR 5564, Grenoble, F-38041, France; E-Mail: jean-paul.laurent@ujf-grenoble.fr

**Keywords:** dielectric constant, water content measurement, rammed earth, cob, hygrothermal behavior, full scale experiments

## Abstract

Rammed earth is a sustainable material with low embodied energy. However, its development as a building material requires a better evaluation of its moisture-thermal buffering abilities and its mechanical behavior. Both of these properties are known to strongly depend on the amount of water contained in wall pores and its evolution. Thus the aim of this paper is to present a procedure to measure this key parameter in rammed earth or cob walls by using two types of probes operating on the Time Domain Reflectometry (TDR) principle. A calibration procedure for the probes requiring solely four parameters is described. This calibration procedure is then used to monitor the hygrothermal behavior of a rammed earth wall (1.5 m × 1 m × 0.5 m), instrumented by six probes during its manufacture, and submitted to insulated, natural convection and forced convection conditions. These measurements underline the robustness of the calibration procedure over a large range of water content, even if the wall is submitted to quite important temperature variations. They also emphasize the importance of gravity on water content heterogeneity when the saturation is high, as well as the role of liquid-to-vapor phase change on the thermal behavior.

## 1. Introduction

The development of earth based buildings is of concern in the actual context of sustainable development, energy consumption and greenhouse gas reduction [1]. Indeed, industrial materials, such as concrete, are major energy consumers during their production and implementation (embodied energy) while their recycling is not always operational [2,3]. The major asset of earth lies in the fact that it is a local material that can be taken and used immediately on the construction site or nearby and does not require industrial processing [4]. It is not a renewable but a reusable material: it requires no treatment to be reused and therefore has a very low impact in terms of energy use [5]. In addition, earth is known to have moisture buffering and temperature controlling properties [6,7]. This is due to the microstructure of the earth, which enables hydric exchanges between the environment and water molecules on the pore surfaces through condensation/evaporation and sorption/desorption phenomena [8]. This affinity with water molecules also significantly impacts mechanical behavior of earth materials. For example, the decrease in strength with moisture, which is well known in soil mechanics [9,10] has been recently demonstrated for rammed earth [11].

Consequently, it appears that the liquid water content of a rammed earth wall is a key parameter in order to understand the behavior and the strength of this material.

In normal conditions, the gravimetric water content of an earth wall (e.g., ratio between water and solid masses) is between 0.5% and 3%. However, when the walls are submitted to pathologies like capillarity (due to water ingress from the saturated soil of the foundation through the basement for example), their water content can drastically increase, up to values close to saturation. This situation cannot be neglected, especially when we consider vernacular buildings. In addition, the monitoring of the water content during the drying stage of the wall, just after its manufacture, is of the utmost importance. It indeed determines the mechanical stability throughout the construction, the risk of damage due to the frost action at early ages, and also significantly impacts the date at which the building can be delivered. Consequently, a proper assessment of an earth wall requires an accurate measurement of its water content whatever its saturation state.

It is however impossible to have a direct access to the mass variation of an *in-situ* wall to measure its water content. It is needed to estimate it indirectly from the non-destructive measurement of physical values which are very sensitive to the presence of water, like the dielectric constant [12], the velocity of ultrasonic waves propagation [13], or the relative humidity [14]. This latter can be easily measured using electronic RH/temperature sensors, and its accuracy to estimate the water content on straw bale walls has already been demonstrated [15]. The relative humidity is linked to the water content by the sorption-desorption curve, which is a routine characterization test for porous materials. However, this relation is accurate only if the in-pore water remains in the hygroscopic domain, which commonly ranges between 0% and 5% of gravimetric water content for rammed earth [16]. In addition, a hysteresis is commonly observed between the sorption and the desorption branches of the curve. This will then induce a loss in the results accuracy if the wall is submitted to repeated wetting-drying cycles of different magnitudes. This method is thus not suited to the present study.

Among the alternative solutions, the use of TDR (Time-Domain Reflectometry) probes, commonly utilized to estimate the *in-situ* soil water constant, is especially appropriate. Indeed, these probes are known to be robust, not expensive, and easy to implement. In addition, they allow measuring the water content of the material whatever its saturation state. These sensors are based on the measurement of the soil dielectric constant, which significantly changes with water content. This is due to the dielectric constant of pure water, equal to 80 at 20°C [17], which is far greater than that of air (=1), and of the solid matrix of a porous medium (up to 14 for clay minerals [18]).

Several authors have proposed studies on mineral soils containing organic matter [19], on clay loam [20,21], on silty granulates [22] or on carbonates [23]. But the deriving relations between the dielectric constant and its water content are unique to these particular materials and cannot be directly used for earth. In addition they are valid only at a given temperature. [24,25] which is problematic in the case of on-site measurement on building walls where considerable fluctuations in temperature occurs.

**Figure 1.**
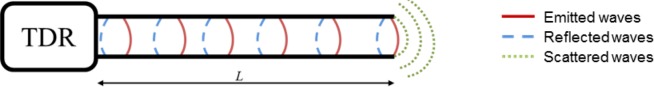
Operating principle of a TDR (Time-Domain Reflectometry) probe.

Moreover, a TDR probe does not directly measure the dielectric constant of the material but the travel time () for the reflection of an electromagnetic wave between two conductive rods (waveguide) of length (*L*) (Figure 1). The relation between the time travel and the dielectric constant is (demonstrated in the Appendix):

$${\varepsilon^{\prime }}_{r}={\left(\frac{c_{0}\tau }{2L}\right)}^{2}-{\left(\frac{\sigma }{\omega \varepsilon_{0}}\right)}^{2}{\left(\frac{L}{c_{0}\tau }\right)}^{2}$$ (1)

where *c*_0_ is the speed of light in vacuum (2.9979 × 10^8^ m·s^−1^); σ the electrical conductivity (S·m^−1^) of the material; ω the angular frequency (s^−1^) and ε_0_ the permittivity of free space (8.85 × 10^−12^ F·m^−1^).

For non-ferromagnetic and non-clay soils the second term in Equation (1) is negligible, which provides a direct relationship between the dielectric constant and the travel time. Regarding material such as rammed earth (or cob), which can contain up to 20% of clay [26], the influence of the electrical conductivity of the medium is not negligible, especially for large amounts of water [25]. In this case, the travel time of the wave no longer provides access to the dielectric constant, but provides an apparent physical quantity called “apparent permittivity” $\varepsilon^{\prime }{}_{a}$ [27], which is a function of the electrical conductivity of the media, and the angular frequency of the signal, both are highly dependent on the saturation ratio. In addition most of the TDR probes do not allow the simultaneous measurement of the travel time and the electrical conductivity of the medium.

Consequently, the measurement of the in-situ water content within earth walls remains an issue that must be solved to have a proper assessment of the earth buildings sustainability.

In this context, the aim of this paper is to propose a simple calibration procedure, which requires minimum measurement points, feasible on site, and adaptable to different types of TDR probes, regardless of whether they provide access to the value of the electrical conductivity.

Note that, the studied material is rammed earth, but this method can be extended to another construction technique like cob, since it is also a stack of soil layers. The only limitation for this method is the amount of clay present in the earth, since clay is a highly conductive material [27].

After a brief description of the probes and materials, the experimental protocol and sensor calibration methodology will be presented. This protocol is then validated on a drying test of earth blocks, and used to estimate the water content of a metric sized rammed earth wall instrumented with six probes.

## 2. Probes and Material Characteristics

### 2.1. Description of the Probes

Two particular types of TDR probes are used: the CS650 which gives access to the electrical conductivity, and the CS616 which does not. Both probes are manufactured by Campbell Scientific and are widely used. Note that, the methodology presented can easily be extended to other types of sensors based on the dielectric permittivity measurement of the medium (other types of TDR probes but also capacitive probes).

#### 2.1.1. CS616

The CS616 has been developed by Anderson and Campbell and operates in the time domain [28]. It is composed of a resin block of dimensions 63 mm × 85 mm × 18 mm and two stainless steel wave guides rods of dimension 300 mm in length, 3.2 mm in diameter and 32 mm spaced, along which an electromagnetic wave is emitted.

The CS616 calculates the travel time τ_m_ by measuring the number of reflections per second. τ_m_ is divided by a scaling factor so that the datalogger can record the data. The measured travel time of the wave is [28]:

$$\tau =\frac{1}{2}(\frac{\tau_{m}}{S_{f}}-2t_{d})$$ (2)

where *S_f_* (=1024) is a scaling factor; τ_m_ is the travel time provided by the probe (μs) and *t_d_* is the delay-time (= 5.5 × 10^−9^ s).

The CS616 does not allow access to the electrical conductivity of the material or to the angular frequency. Thus, as already noted in [27,29] and as mentioned in the introduction, it is not directly the permittivity which is estimated, but an apparent permittivity equal to:

$$\varepsilon^{\prime }{}_{a}={\left(\left(\frac{\tau_{m}}{S_{f}}-2t_{d}\right)\frac{c_{0}}{4L})\right)}^{2}$$ (3)

In Equation (3), is the effective length of the rods which is equal to 0.26m, according to the study of [30] on these sensors.

Note that, according to the manufacturer [31], if the sensor accuracy is ± 2.5% for σ < 0.5 dS·m^−1^ and a dry density of 1.55, a significant difference between $\varepsilon^{\prime }{}_{a}$ and $\varepsilon^{\prime }{}_{r}$ appears for values of σ above 0.5 dS·m^−1^.

#### 2.1.2. CS650

The CS650 works on the same principle of reflectometry and is physically identical to the CS616. This probe converts directly the measured response into a digital signal and sends it to the datalogger through a SDI-12 protocol.

The CS650 determines the electrical conductivity by measuring the attenuation of the signal. It also measures the angular frequency—but does not give access to it—and automatically corrects the measured travel time (see Equation (2)). It gives access to the “real” dielectric permittivity $\varepsilon^{\prime }{}_{r}$ and not to $\varepsilon^{\prime }{}_{a}$.

### 2.2. Material Characteristics and Sample Preparation

**Figure 2.**
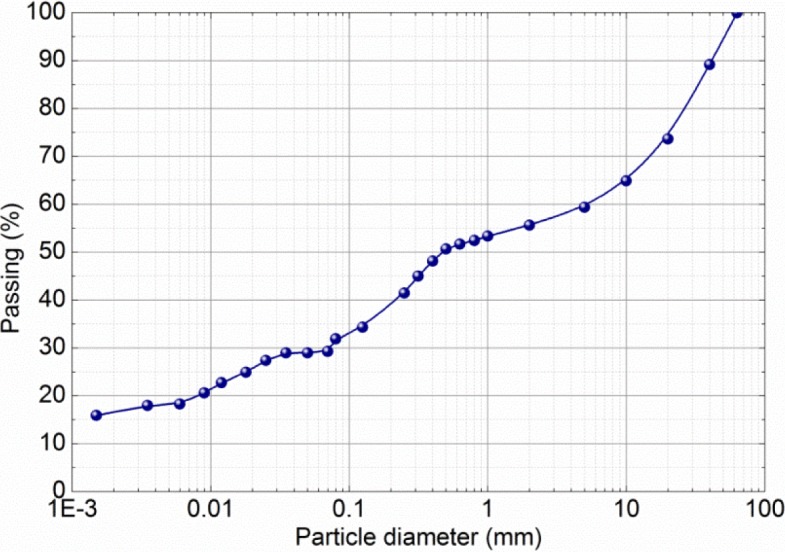
Particle-size distribution.

The studied material comes from the construction site of a rammed earth house built in 2011 in France. It is a local earth mixed with 2.5% (in dry weight) of NHL5 lime. Its characteristics are presented in Table 1 and particle-size distribution (following the NF P 94-056 and NF P 94-057 standards [32,33]) in Figure 2.

As mentioned earlier, it is not possible to estimate directly the *in-situ* water content in the wall of the building. The first step for calibration is therefore to reproduce a small size sample, which can be easily transportable to be weighed, but is also representative of the relation between the water content and the dielectric constant of the rammed earth wall. For a given material this relation strongly depends on the volume and geometry of the porous network [34]. In the case of rammed earth, the pore volume can be controlled by the dry density, while for the same density, the geometry of the pore network will be affected by the compaction technic and the water content during compaction [35].

Thus in order to calibrate the probes we inserted them into two blocks of compacted earth of 45 cm × 15 cm × 9 cm dimensions with the dry density of 1730 kg·m^−3^ during the blocks manufacture. Probes are placed between two layers surrounded by one centimeter of loose material to avoid large granulates which could damage it. The blocks were made with a pneumatic rammer by the mason who built the house where the earth comes from and with gravimetric water contents (ratio between the mass of water and the mass of dry earth), as close as possible: = 20.1% for the block containing the CS616 and = 19.6% for the block containing the CS650.

This gravimetric water content is two times higher than that normally used in rammed earth and cob [36]. This is mainly due to the presence of 2.5 wt% lime NHL5 [37].

Let us emphasize here that the average dielectric constant of the material depends on the volume fraction of the several phases that form the porous material [34]. Thus, in the following, we will rather use the volumetric water content θ (= volume of water/volume of the block), instead of the gravimetric water content *w* (= mass of water/mass of dry earth). The link between them is:

$$\theta =d_{s}w$$ (4)

where *d_s_* is the ratio between the dry density of the sample and the water density. Thus considering *d_s_* = 1.73, the initial volumetric water content for the blocks containing the CS616 and the CS650 are respectively equal to 34.8% and 33.9%.

## 3. Calibration of the Probes

### 3.1. Calibration of the Probes

The CS650 directly provides a volumetric water content value according to the relation proposed by Topp for generic soil containing minerals [19]. However, this relation is not suited for rammed earth and cob. The CS616, for its part, does not provide direct access to the dielectric permittivity but to an apparent permittivity (see Equations (1–3)).

In this context, the approach initially developed by [18] is followed. It consists of assuming that the measured permittivity (e.g. either $\varepsilon^{\prime }{}_{r}$ measured by the CS650 or $\varepsilon^{\prime }{}_{a}$ measured by the CS616) can be expressed as a bilinear function of volumetric water content of the material (θ) and its temperature (*T*, in °C):

$$\varepsilon_{r}^{\prime }=AT\text{θ}+BT+C\text{θ}+D;\text{for}\;\text{CS}650$$ (5)

$${\text{ε}}^{\prime }{}_{\text{a}}=A^{\prime }T\text{θ}+B^{\prime }T+C^{\prime }\text{θ}+D^{\prime };\prime \text{for}\;\text{CS}616$$ (6)

where *A*, *B*, *C*, *D* and *A*′, *B*′, *C*′, *D*′ are respectively the calibration coefficients for the CS650 and the CS616 probes. Let us note that the applicability of this relation was already verified for freezing-thawing clay-based materials [38]. Its major advantage is that it uses only four parameters (instead of ten for the Topp’s expression). Thus, the calibration coefficients are directly accessible via the measurement of the evolution of the permittivity with temperature for two determined volumetric water contents.

Consequently, to calibrate the probes, blocks at given volumetric water content (0%, 7.1% and 13.9% for the CS650 and 0%, 9.8% and 14.8% for the CS616) are wrapped in plastic film and put in an oven at a controlled and fixed temperature until the stabilization of the probe output signal. The temperature homogeneity is controlled by comparing the temperatures inside the oven and inside the sample. An additional volumetric water content is used, three instead of the two which are necessary in order to estimate the error on the coefficients. Results are reported in Table 2 for the CS650 and in Table 3 for the CS616.

The resulting calibration coefficients are: *A* = 0.84 ± 0.07; *B* = 0.014 ± 0.01; *C* = 47.07 ± 1.79; *D* = 2.41 ± 0.01; for the CS650 and to *A*′ = 1.38 ± 0.18; *B*′ = 0.014 ± 0.01; *C*′ = 51.06 ± 4.69; *D*′ = 2.40 ± 0.01 for the CS616.

### 3.2. Verification of the Calibration

**Figure 3:**
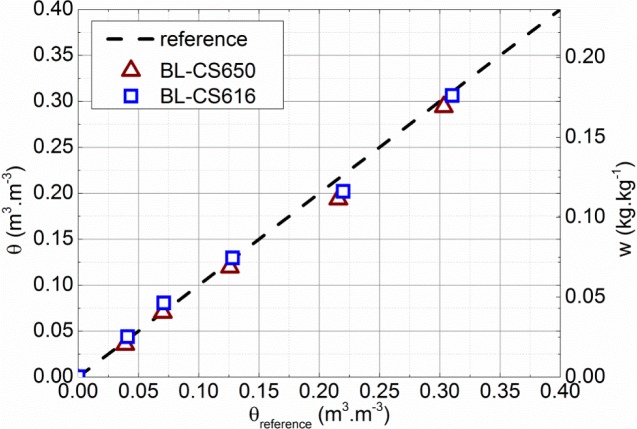
Comparison between the volumetric water contents obtained by weighing (θ_reference_) and from the probes CS650 (θ_BL−CS650_) and CS616 (θ_BL−CS616_) calibrated with the bilinear relation.

The accuracy of the calibration procedure using the bilinear relations (5), and (6) is checked by performing a drying test on two rammed earth blocks with the same dimensions and manufacturing process as those used for calibration and respectively instrumented by a CS616 and a CS650. Immediately after their manufacture, the blocks are weighed and wrapped in a sealed plastic film. This step is necessary to homogenize the water content throughout their volume. The probes are then connected to the datalogger and a point is recorded every fifteen minutes. Once the signal returned by the probes is stabilized (*i.e.* the homogeneous water content in the volume) the blocks are weighed to determine the moisture content and then are dried for five days in a room controlled at 20 °C. They are then wrapped again in a sealed plastic film until homogenization of the moisture content throughout their volume. This procedure is repeated until the mass variation during the drying stage becomes negligible. The blocks are finally oven dried at 50 °C to reach the reference dried state (θ=0). The drying is done concurrently and under the same conditions for both blocks. During the whole test the volumetric water content is monitored by both sample weighing (using a dry density of 1730 kg/m^3^) and probe measurements. The comparison between these two values for both probes is reported in Figure 3.

An average difference lower than 0.5% between the measured volumetric water content by weighing and the estimated one from the probes is then observed, which is broadly acceptable. Let us highlight that the calibration procedure presented in this paper allows the use of CS616 sensors, even if they are not recommended for these kinds of materials.

## 4. Application on the Drying of a Rammed Earth Wall

**Figure 4.**
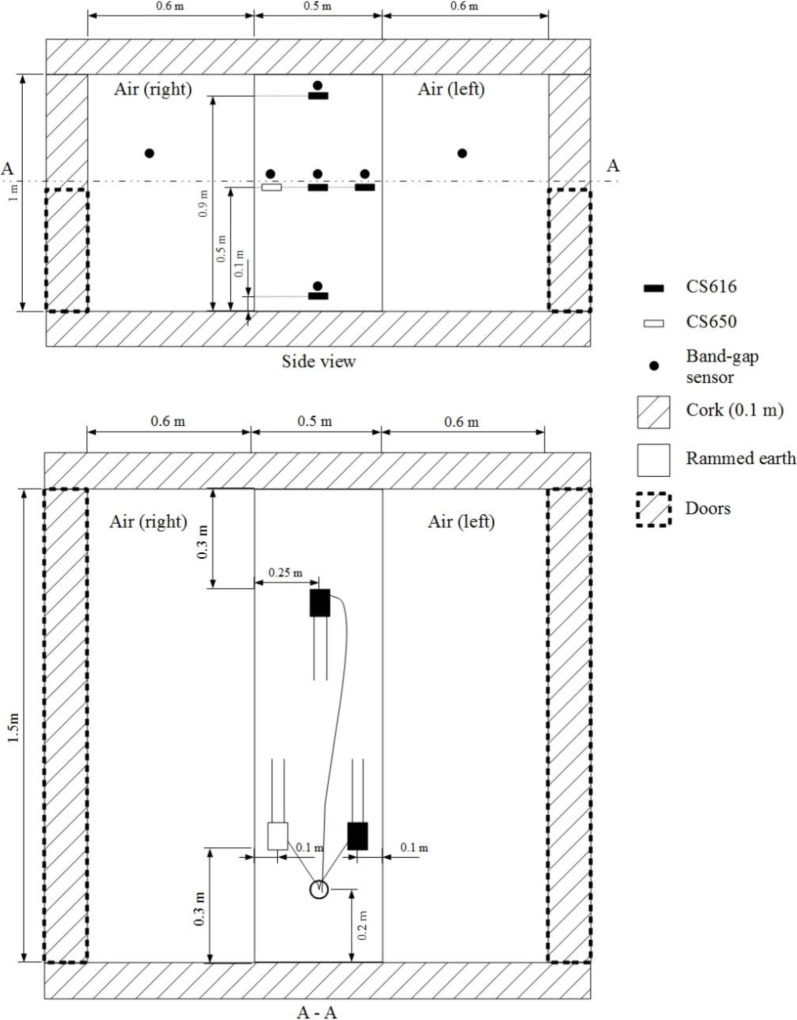
Side view and top view of the implementation of CS616 and CS650 probes in the rammed earth wall placed in a sealed box.

The calibration procedure was used to study the hydric behavior of a full scale earth wall of dimensions 1 m ×1.5 m × 0.5 m equipped with five TDR probes and five band-gap sensors. Three CS616 are positioned at the mid thickness and at three different heights: 0.9 m (top), 0.5 m (middle) and 0.1 m (bottom). At 0.5 m height, one CS616 and one CS650 are installed at 0.1 m of both faces of the wall. At this particular height, according to the manufacturer, probes are placed in a staggered configuration to prevent any electromagnetic interference. To avoid any damage, the probes are surrounded by loose material before ramming. In addition, three nails are used to keep the probes in their initial position. The temperature is measured by band-gap sensors placed just above each TDR probe. Two other band-gap sensors are also installed on the right and left ambiances of the box. This implementation is illustrated in Figure 4.

**Figure 5.**
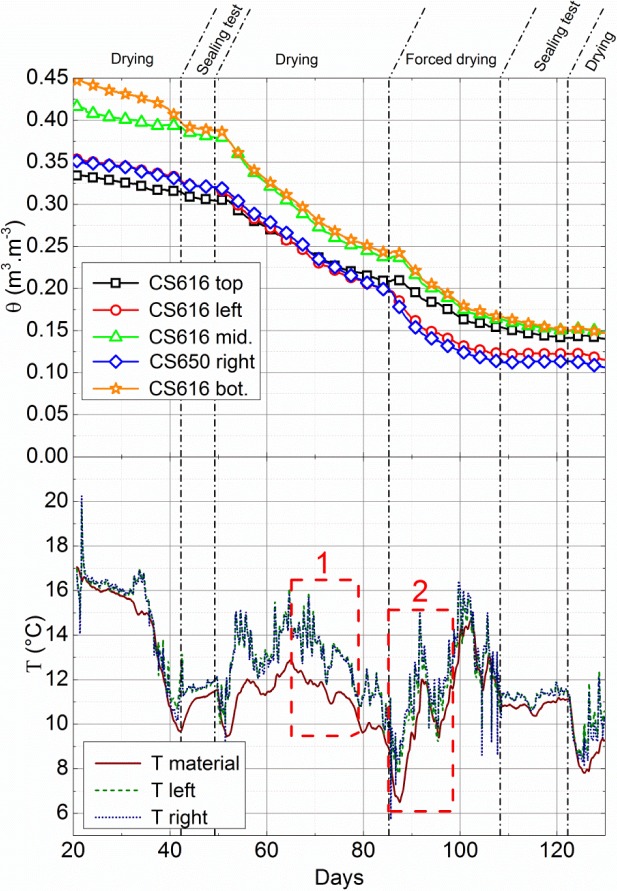
Evolution of (up) volumetric water content and (down) temperature of the wall.

The wall and the blocks used for the calibration were built by the same mason, using the same protocol and with a similar gravimetric water content (*w*_wall_ = 18.9%). Probes are placed during the manufacture and in a staggered row to prevent any electromagnetic interference between them. For technical reasons, after its manufacture, the wall is protected by a waterproof plastic film to slow its drying over 22 days. It is then placed in a box, designed in the laboratory, with 10 cm thick cork for insulation (Figure 4). For 130 days, one data per hour is recorded. The natural drying periods (opening of the “removable walls” of the box) are interspersed by periods of sealing tests of the boxes: from the 44th to 51st day and from the 110th to 124th day. Forced drying experiments are also performed by forced air circulation with a fan on each surface. The corresponding dates are from 87th to 110th day. During the whole test, moisture and temperature conditions are identical on both sides of the walls. The volumetric water contents measured by the probes and the temperature within the box and the wall are reported in Figure 5.

### 4.1. Verification of the Accuracy of the Probes

The accuracy of the calibration procedure is estimated from the comparison between the volumetric water content measured by the CS616 and CS650 probes, located respectively at the left and right sides. Indeed, because the drying conditions on both sides of the walls are identical, the two probes should then provide the same value. The error in the volumetric water content can thus be quantified by:

$$\Delta_{\theta }=\frac{\left|\theta_{CS616}-\theta_{CS650}\right|}{\theta_{CS616}}$$ (7)

**Figure 6.**
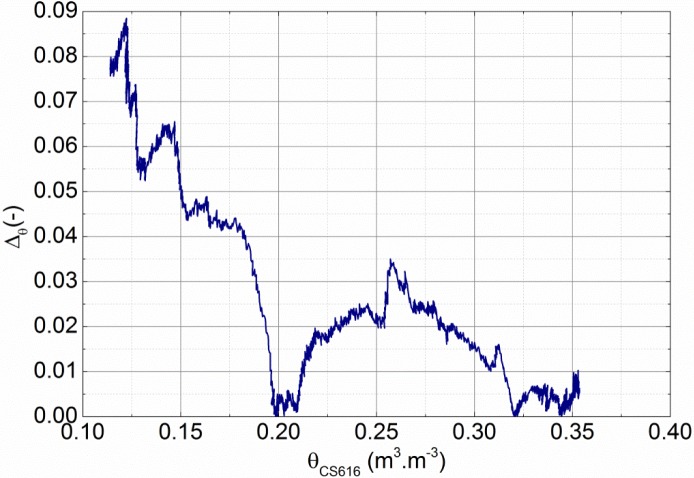
Deviation Δ_θ_ on the values of volumetric water content between the CS616 and CS650 probes located respectively on the left and right sides 50cm high.

The evolution of Δ_θ_ with volumetric water content is reported in Figure 6. It shows a small average error of 3.3% with a peak value of 9% for θ = 12.4%. The results remain then close, while the test conditions are not the same as the ones used for the calibration. In addition, as sketched in Figure 5, the wall is submitted to temperature variations (from 7 to 20 °C). However, it does not seem to disturb the measured value of volumetric water content, while the impact of temperature on the measured dielectric constant is different for the two probes. It gives some confidence of the accuracy of calibration.

### 4.2. Transient Behavior

The first values were recorded just after the removal of the waterproof plastic film (e.g., 22 days after the manufacturing of the walls). Assuming that the effect of gravity is negligible and the plastic film is completely sealed, all the probes should provide the same value. It is however not the case. A water content gradient is measured between the center and the sides of the wall. It must be due to the fact that the drying of the wall from its sides is faster than the homogenization of the water content through the wall thickness (which is driven by water transport processes within the porous network of the material). The initial difference could then be explained by a defect in wrapping which would therefore slow the drying instead of stopping it during the storage phase of 22 days.

However, the magnitude of this difference (up to 7% between the sides and the center of the wall) is surprisingly high. It suggests a very small water relative permeability of the material. This fact is also observed during the sealing tests (from 44th to 51st day and from 110th to 124th day). Indeed the closure of the box doors leads to a fast stabilization of the volumetric water content measured by the probes located on the right and left sides while the volumetric water content in the center of the wall (top, middle and bottom CS616) still decreases. As drying almost stops during this stage, this volumetric water content variation is mainly due to liquid water transport from the core to the sides of the wall. However, even if these periods are quite long (respectively 7 days and 14 days), they are not sufficient to reach an equilibrium state (e.g. stabilization of the volumetric water content in the center of the wall).

Thus, this first observation underlines that a variation of volumetric water content is expected within the thickness of a rammed earth wall. Accounting for the great dependency of the material properties with water content, this heterogeneity may have some impact on its global behavior and should be taken into account.

### 4.3. Effect of Gravity

**Figure 7.**
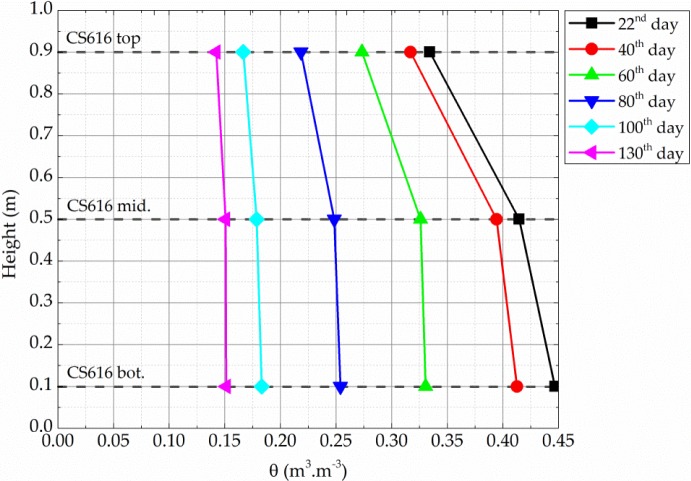
Volumetric water content depending on the height in the wall.

In addition, a decrease with height in the measured volumetric water content is observed (*i.e.*, θ*_top_* <θ*_middle_* <θ*_bottom_*). As sketched in Figure 7, this difference decreases with time, and the volumetric water contents measured by the three probes converge to a mean value of 6.8% ± 0.15%.

Actually, when the wall is close to saturation, the capillary suction in the wall—*i.e.* water depression compared to air, due to the meniscus formed by the water/air interface which increases as the water content decreases [9,18]—is not sufficient to counter the gravity effect. Consequently, a flow occurs from the upper to the lower part of the wall.

Figure 7 therefore highlights the importance of taking into account the effect of gravity, at least when earth buildings are submitted to quite high water contents (>10% gravimetric water content). This condition happens during the manufacture of the wall, at early ages (during its original drying, that lasts a few months according to Figures 5 and 7), and when the wall is submitted to some pathologies like capillarity, which all represent significant economical stakes for the vernacular architecture throughout the world.

Conversely, when the water content decreases, the capillary suction forces becomes predominant against gravity forces, which leads to the same moisture content throughout the height of the wall (Figure 7).

### 4.4. Hygrothermal Behavior

Let us now focus on the coupling between water content and temperature measurements. When the volumetric water content variation remains quite small (e.g. last sealing tests or end of the drying curve), no significant differences are observed between the temperature within the box and the one within the wall. However, as sketched in Figure 2, during the period where the water content significantly decreases (from 51st to 110th day), a significant decrease of temperature inside the wall is observed. A simple way to explain this temperature variation is to use the thermal equation in its uni-dimensional form for porous media with in-pore water phase change [39]:

$$\rho C\frac{\partial T}{\partial t}=\lambda \frac{\partial^{2}T}{\partial x^{2}}+\dot{m}L_{V}$$ (8)

where ρ*C* is the average heat capacity of the material, and λ is its average thermal conductivity. They both vary with water content and temperature. However, it is assumed here that they are constant and respectively equal to 1500 J·m^−3^·K^−1^ and 1.6 W·m^−1^·K^−1^, which are their estimated values at 20 °C with θ = 20%.

Finally, *L_V_* stands for the latent heat of evaporation/condensation and $\dot{m}$ is the rate of water mass which evaporates (resp. condensates) per unit of material volume. The last term of this equation is positive during condensation and negative during evaporation, and can thus be the cause of the wall temperature decrease during the drying stage. However, the quantification of this effect is not so simple. Indeed, a smaller decrease in temperature is observed during the forced drying period (from 87th to 110th day) than during the second drying stage (from 51st to 87th day) while the volumetric water content variation rate is similar for the both stages (0.37%/day for the forced drying test between 90th and 98th day and 0.33%/day for the natural drying test between 70th and 78th day).

To understand this behavior, the partial differential Equation (8) is solved with COMSOL Multiphysics^®^ using the PDE module. The simulations are made for a 1D geometry of length L=0.5 m (*x* direction). It represents a lateral cross section of the tested wall.

Boundary conditions of the simulations are set according to measurements within both sides of the insulated box. Thus, thermal exchanges on *x* = 0 and *x* = e are calculated by means of the following equation:

$$\lambda \frac{\partial T}{\partial x}=h(T_{\text{box}}-T_{S})$$ (9)

where *T*_box_ and *T_s_* are temperatures of respectively the ambient air in the box and on the side of the wall; while *h* is the heat transfer coefficient. It is assumed to be equal to 15 W·m^−2^·K^−1^ during the drying stage and equal to 250 W·m^−2^·K^−1^ during the forced drying stage, which are common values for resp. natural and forced convection conditions [40].

Calculation are made either with *L_V_* = 2260 kJ·kg^−1^ (calculation with latent heat) and *L_V_* = 0 kJ·kg^−1^ (calculation without latent heat). Finally, $\dot{m}$ is directly derived from the volumetric water content measurements through the relation:

$$\dot{m}=\rho_{L}\frac{\partial \theta }{\partial t}$$ (10)

where ρ*_L_* is the density of water. Let us remark that, strictly speaking, the variation of water content of the material is due to phase change and water transport. Thus, a precise evaluation of $\dot{m}$ requires a fully coupled hygrothermal model. However, as previously discussed, the water transport remains quite slow, and consequently, at first order, we may assume that the relation (10) can be used.

**Figure 8.**
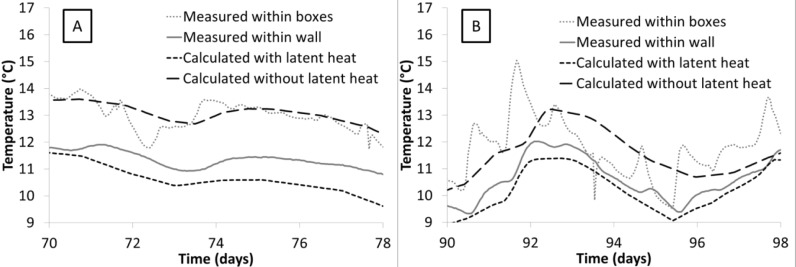
Focus on the temperature within the wall and within the boxes during (**A**) a drying stage and (**B**) a forced drying stage.

The comparison between the calculated temperatures with the measured one within the wall at *x* = 0.25 m is reported in Figure 8.

First, for both simulations (e.g. natural drying and forced drying), the calculated values with latent heat are very close to measured values, which is not the case for simulations without latent heat. They however tend to overestimate the latent heat effect; this may be due to the use of Equation (10), which overestimates the evaporation rate by neglecting the effect of water transport on water content variations. This result is, by itself, another proof of the accuracy of the volumetric water content measured by the probes. Indeed, the temperature decrease due to phase change is very sensitive to the amount of water that evaporates, and thus to the measurement given by the probes.

The second observation is the strong reduction of impact of the phase change phenomenon on the wall temperature when the forced drying conditions are considered. Actually, in this case, due to the high value of the heat transfer coefficient, the heat consumption by the liquid to vapor phase change is more than compensated by the incoming heat flow at the wall surface.

Then, phase change phenomena appear to impact the thermal behavior of earth materials, and must be considered for a proper thermal assessment of earth buildings. However, the magnitude of this impact strongly depends on the wall solicitation (external/internal convections, wrapped), and should therefore require the use of a coupled hygrothermal model for its accurate evaluation.

## 5. Conclusion

A calibration method of rammed earth and cob *in-situ* volumetric water content measurement with TDR sensors is presented. The main asset of this method is to allow a simple and fast calibration using only four parameters from the measurement of the evolution of the permittivity with temperature for two determined volumetric water contents. The accuracy of this methodology was tested on drying experiments of two rammed earth blocks.

This calibration procedure was then used to measure the volumetric water content within a metric rammed earth wall instrumented with six TDR probes, and submitted to natural drying, forced drying and wrapped conditions with temperature variations.

For a given height, the data from the probes on the left and right sides are almost identical. Knowing that the two probes operate differently and have been calibrated separately, this result confirms the robustness of the calibration protocol.

The effect of gravity is observed at the beginning of the drying process and could involve an *ad-hoc* modeling. However, when the wall is drying, the volumetric water content between the top and bottom tends to homogenize after a few months. It highlights the importance of taking into account the effect of gravity to properly simulate the behavior of earth buildings during their manufacture, at early ages or when submitted to pathologies like capillarity.

Finally, a decrease in temperature in the wall is observed during the drying stages. This effect, due to the latent heat of evaporation, was successfully linked to the measurements of volumetric water content variations through a uni-dimensional simulation. It emphasizes the importance of the coupled hygrothermal processes for an accurate assessment of the thermal behavior of earth buildings.

In France, an inhabited rammed earth house sees gravimetric water content varied from 0.5% to 3%. The sensors studied in this paper seem to have sufficient accuracy to measure this type of variation. A new rammed earth house was built in France and equipped with CS616 probes to characterize its hygrothermal behavior. The calibration procedure performed in this paper will enable the water content of the new construction to be measured over time.

## Figures and Tables

**Table 1.** Characteristics of the manufactured earth.

Property	Symbol	Value	Units	Standard deviation
Dry density	ρ	1730	kg·m^−3^	0.04
Porosity	Φ	0.347	m^3^·m^−3^	0.015
Dry electrical permittivity (20°C)	$\varepsilon^{\prime }{}_{r}$	2.5	–	–
Clay content (<2 μm, NF P 94-057)	–	16	%	–
Manufacturing gravimetric moisture content	w*_ref_*	0.183	kg·kg^−1^	0.009

**Table 2.** Variation of $\varepsilon^{\prime }{}_{r}$ with temperature at three volumetric water contents with the CS650.

Water content, θ (m^3^·m^−3^)	Temperature, *T* (°C)	Dielectric permittivity, $\varepsilon^{\prime }{}_{r}$ (1)
0	13.5 50	2.6 3.1
0.071	20 50	7.2 9.5
0.139	20 50	11.6 15.3

**Table 3.** Variation of $\varepsilon^{\prime }{}_{a}$ with temperature at three volumetric water contents with the CS16.

Water content, θ (m^3^·m^−3^)	Temperature, *T* (°C)	Apparent permittivity, $\varepsilon^{\prime }{}_{a}$ (1)
0	13.5 50	2.6 3.1
0.098	20 50	10.7 14.5
0.148	20 50	14.2 20.7

## Appendix

The principle of TDR measurement is of wave propagation in an absorbing medium. The solution of Maxwell’s equation for the electric field E in a z direction is:

$$E=E_{0}e^{-i(\omega t-k^{*}z)}$$ (A1)

where ω is the angular frequency and *k**^*^* the complex wave number defined as:

$$k^{*}=\frac{\omega }{c_{0}}\sqrt{\varepsilon_{r}^{*}}$$ (A2)

where *C*_0_ is the speed of light in vacuum (2.9979 10^8^ m·s^−1^) and $\varepsilon_{r}^{*}$ the dielectric permittivity of the medium.

If *k**^*^* and $\varepsilon_{r}^{*}$ are defined as:

$$k^{*}=k^{\prime }+i\;k^{\prime \prime }$$ (A3)

$${\text{ε}}_{r}^{*}=\varepsilon^{\prime }{}_{r}+i\;\varepsilon_{r}{}^{\prime \text{​}\prime }$$ (A4)

Thus:

$$\sqrt{\varepsilon_{r}^{*}}=\frac{1}{\sqrt{2}}\left(\sqrt{\left(\sqrt{{\left({\text{ε}}^{\prime }{}_{r}\right)}^{2}+{\left({\text{ε}}^{\prime \prime }{}_{r}\right)}^{2}}+{\text{ε}}^{\prime }{}_{r}\right)}+i\sqrt{\left(\sqrt{{\left({\text{ε}}^{\prime }{}_{r}\right)}^{2}+{\left({\text{ε}}^{\prime \prime }{}_{r}\right)}^{2}}-{\text{ε}}^{\prime }{}_{r})\right)}\right)$$ (A5)

and the imaginary and real parts of *k**^*^*:

$$k^{\prime }=\frac{\omega }{\sqrt{2}c_{0}}\sqrt{\sqrt{{\left(\varepsilon^{\prime }{}_{r}\right)}^{2}+{\left(\varepsilon^{\prime \prime }{}_{r}\right)}^{2}}+\varepsilon^{\prime }{}_{r}}$$ (A6)

$$k^{\prime \prime }=\frac{\omega }{\sqrt{2}c_{0}}\sqrt{\sqrt{{\left(\varepsilon^{\prime }{}_{r}\right)}^{2}+{\left(\varepsilon^{\prime \prime }{}_{r}\right)}^{2}}+\varepsilon^{\prime }{}_{r}}$$ (A7)

The electric field can then be written as:

$$E=E_{0}\text{ exp}(-i(\omega t-k^{\prime }z-ik^{\prime \prime }z))=E_{0}\text{ exp}(-k^{\prime \prime }z)\text{ exp}\;\;(-i(\omega t-k^{\prime }z))$$ (A8)

$$E=E_{0}e^{\left(-\frac{\omega z}{\sqrt{2}c_{0}}\sqrt{\sqrt{{\left(\varepsilon^{\prime }{}_{r}\right)}^{2}+{\left(\varepsilon^{\prime \prime }{}_{r}\right)}^{2}}-\varepsilon^{\prime }{}_{r}}\right)}e^{-i\omega \left(t-\frac{z}{\sqrt{2}c_{0}}\sqrt{\sqrt{{\left(\varepsilon^{\prime }{}_{r}\right)}^{2}+{\left(\varepsilon^{\prime \prime }{}_{r}\right)}^{2}}+\varepsilon^{\prime }{}_{r}}\right)}$$ (A9)

$$E=A(z)e^{-i\omega \left(t-\frac{z}{c_{f}}\right)}$$ (A10)

where is the amplitude of the wave and the wave velocity in a non-absorbent material. The travel time of the wave through the material thickness is the smallest value of t,Δ*t*, to cancel the imaginary part of E, which is:

$$\Delta t=\frac{\Delta z}{c_{f}}=\frac{\Delta z}{\sqrt{2}c_{0}}\sqrt{\sqrt{{\left(\varepsilon^{\prime }{}_{r}\right)}^{2}+{\left(\varepsilon^{\prime \prime }{}_{r}\right)}^{2}}+\varepsilon^{\prime }{}_{r}}$$ (A11)

$$\Rightarrow 2{\left(\frac{c_{0}\Delta t}{\Delta z}\right)}^{2}=\sqrt{{\left(\varepsilon^{\prime }{}_{r}\right)}^{2}+{\left(\varepsilon^{\prime \prime }{}_{r}\right)}^{2}}+\varepsilon^{\prime }{}_{r}$$ (A12)

Then the real part of the dielectric permittivity $\varepsilon^{\prime }{}_{r}$ can now be written as:

$$\varepsilon^{\prime }{}_{r}={\left(\frac{c_{0}\Delta t}{\Delta z}\right)}^{2}-{\left(\frac{\Delta z}{2\Delta t\;c_{0}}\right)}^{2}{\left(\varepsilon^{\prime \prime }{}_{r}\right)}^{2}$$ (A13)

The length of the rods of the probe is noted L and then is equal to:

Δz=2L (A14)

If the losses due to dielectric relaxation are neglected (small compared to t), then:

$$\varepsilon^{\prime \prime }{}_{r}=\frac{\sigma }{\omega \varepsilon_{0}}$$ (A15)

and this leads to:

$$\varepsilon^{\prime }{}_{r}={\left(\frac{c_{0}\Delta t}{2L}\right)}^{2}-{\left(\frac{L}{c_{0}\Delta t}\right)}^{2}{\left(\frac{\sigma }{\omega \varepsilon_{0}}\right)}^{2}$$ (A16)
